# Reduced Ca^2+ ^transport across sarcolemma but enhanced spontaneous activity in cardiomyocytes isolated from left atrium-pulmonary veins tissue of myopathic hamster

**DOI:** 10.1186/1423-0127-16-114

**Published:** 2009-12-29

**Authors:** Yue-Xia Loh, Kuo-Ho Wu, Yao-Chang Chen, Chih-Hsiung Hsu, Jeng Wei, Cheng-I Lin

**Affiliations:** 1 Institute of Physiology, National Defense Medical Center, Taipei, Taiwan, ROC; 2 Graduate Institute of Medical Sciences, National Defense Medical Center, Taipei, Taiwan, ROC; 3 Department of Biomedical Engineering, National Defense Medical Center, Taipei, Taiwan, ROC; 4 Section of Cardiology, Tri-Service General Hospital, National Defense Medical Center, Taipei, Taiwan, ROC; 5 Chiayi Veterans Hospital, Taipei, Taiwan, ROC; 6 Heart Center, Cheng-Hsin General Hospital, Taipei, Taiwan, ROC

## Abstract

**Background:**

Several lines of evidence point to a particularly important role of the left atrium (LA) in initiating and maintaining atrial fibrillation (AF). This role may be related to the location of pulmonary veins (PVs) in the LA. The aim of the present study was to investigate the action potential (AP) and ionic currents in LA-PV cardiomyocytes isolated from Bio14.6 myopathic Syrian hamsters (36-57 week-old) versus age-matched F1B healthy control hamsters.

**Methods and Results:**

Whole-cell patch-clamp techniques were used to record AP in current-clamp mode and ionic currents in voltage-clamp mode. The results obtained show that in both healthy and myopathic LA-PV tissue spontaneously discharging cardiomyocytes can be found, but they are more numerous in myopathic (9/29) than in healthy hamsters (4/42, p < 0.05 by χ^2 ^analysis). Myopathic myocytes have shorter AP duration (APD) with smaller I_Ca,L _and I_NCX _than the healthy control. The currents I_TO_, I_K_, I_K1 _and I_Ca,T _are not significantly different in myopathic versus healthy cells.

**Conclusions:**

Our results indicate that in myopathic Syrian hamsters LA-PV cardiomyocytes are more prone to automatic rhythms. Also, they show altered electrophysiologic properties, which may be due to abnormal Ca^2+ ^channels and may account for contractile dysfunction.

## Introduction

The Biobreeders strain 14.6 myopathic Syrian hamster had been frequently used as an experimental model for the study of congestive heart failure (CHF) [1-3]. It has been proposed that, because of inherited defects in cytosolic calcium (Ca^2+^_i_) reuptake in the sarcoplasmic reticulum (SR) [4,5], myopathic myocytes would be more prone to develop Ca^2+^_i _overload and related triggered arrhythmia [6]. In this animal, the evolution of the disease is characterized by (a) an early stage of cellular necrosis (40 to 60 days), (b) a mid-stage of cardiac hypertrophy and ventricular dilation (100 to 300 days), and (c) an end stage of de-compensated heart failure (>360 days).

Recently, an increased incidence of atrial fibrillation (AF) has been shown in patients with more advanced CHF [7]. In the left atrium (LA), the pulmonary veins (PVs) are an important source of ectopic beats, which frequently initiate paroxysms of AF. These foci respond to treatment with radio-frequency ablation [8,9]. Previous studies in multiple or single cells from dogs [10,11] and rabbits [12,13] have shown that PVs contain cardiomyocytes, which may be either quiescent or show spontaneous activity. However, it is not clear whether CHF enhances the arrhythmogenic activity of PVs.

The aim of the present experiments was to study whether there are alterations in action potential and ionic currents in LA-PV myocytes of Biobreeders Syrian hamsters at mid- and late-stages of cardiac failure (36-57 week-old) compared to healthy hamsters of the similar age. A preliminary report has appeared in abstract form [14].

## Materials and methods

### Isolation of single PV cardiomyocytes

All experiments were performed according to institutional guidelines. Male cardiomyopathic hamsters (Bio14.6) and normal F1B hamsters purchased from Bio Breeders Inc. (Fitchburg, MA, USA) and aged 36 to 57 weeks, were used for the experiments. Hamsters were anesthetized with intraperitoneal injection of sodium pentobarbital (50 mg/kg). A mid-line thoracotomy was quickly performed along with removal of heart and lungs. The PVs were perfused in a retrograde manner via polyethylene tube passed through the aorta and left ventricle into the left atrium. The proximal end of the polyethylene tubing was connected to a Langendorff apparatus for perfusion with oxygenated Tyrode's solution at 37°C. The Tyrode's solution contained (in mmol/L) NaCl 137, KCl 5.4, CaCl_2 _1.8, MgCl_2 _0.5, HEPES 10 and glucose 11 (pH was adjusted to 7.4 with NaOH) for about 15 minutes until efferent fluid was without blood.

The perfusate was then replaced with oxygenated Ca^2+^-free Tyrode's solution containing 1 mg/ml collagenase (Sigma, Type I) and 0.01 mg/ml protease (Sigma, Type XIV) for 30-40 minutes. Afterwards, the heart was washed with oxygenated Ca^2+^-free Tyrode's solution for 10 minutes. After that, the LA-PV area was removed from the heart, cut into fine pieces and gently shaken in 5-10 ml high-K^+ ^storage solution until single cardiomyocytes were obtained.

### Cellular electrophysiology

Only LA-PV cardiomyocytes with clear cross striations obtained from 19 myopathic hamsters and 22 healthy hamsters were used for electrophysiological studies. Action potentials and ionic currents were recorded by means of whole-cell patch-clamp techniques with an Axopatch 1D amplifier (Axon Instruments, CA, U.S.A) as described in detail recently [11,13,15]. The standard pipette solution contained (in mmol/L) KCl 20, K aspartate 110, MgCl_2 _1, EGTA 0.5, Mg_2_ATP 5, Na_2_phosphocreatine 5, LiGTP 0.1, and HEPES 10, adjusted to pH 7.2 with KOH. The standard extracellular solution was the normal Tyrode's solution also used in cell isolation. Ionic currents were recorded in voltage-clamp mode. For the recording of the calcium currents I_Ca,L _and I_Ca,T_, tetraethylammonium (TEA) chloride and CsCl replaced NaCl and KCl, respectively. For I_Ca,T _recording, tetrodotoxin (5 μmol/L) was added to block the fast Na^+ ^current (I_Na_). For K^+ ^current measurement, CdCl_2 _(200 μmol/L) was added to block I_Ca,L_. A 30-ms prepulse from -80 to -40 mV was used to inactivate the sodium channel, followed by a 300-ms test pulses in 10-mV increments to +60 mV. I_K1 _was quantified as 1 mmol/L Ba^2+^-sensitive current.

Action potentials (APs) were recorded in current-clamp mode. The tip potentials were zeroed before formation of the membrane-pipette seal in Tyrode's solution. After rupture, junction potential (8 mV) was corrected for AP recording. A small hyperpolarizing step from a holding potential of -50 mV to a testing potential of -55 mV was used to obtaining the total cell capacitance at the beginning of each experiment. The area under the capacitative current was divided by the applied voltage step to obtain the total cell capacitance. Normally 60-80% of series resistance (R_S_) was electronically compensated. After compensation, the average time constant was 71 ± 6 μs, (cell capacitance 33 ± 2 pF, for 83 cells in healthy group; and 47 ± 3 pF for 67 cells in myopathic group). The average R_S _was 1.8 ± 0.1 MΩ for all cells examined. Currents rarely exceeded 1.2 nA, and the maximal voltage error did not exceed 3 mV.

Voltage command pulses were generated by a 12-bit digital-to-analog converter controlled by pCLAMP software (Axon Instruments). APs were elicited by pulses of 2 ms and 90 mV at a rate of 1 Hz. AP measurements were begun 3 minutes after cell membrane rupture, and the steady-state AP duration was measured at 20% (APD_20_), 50% (APD_50_) and 90% (APD_90_) of full repolarization. Depolarization-induced currents included the transient outward K^+ ^current (I_TO_) and the delayed rectifier outward K^+ ^current (I_K_), and were elicited by applying steps from a holding potential (V_h_) of -40 mV in 10 mV increment up to +60 mV at a frequency of 0.1 Hz.

In recording calcium currents, to minimize the effect of "rundown" [13], the depolarizing steps were applied from a holding potential which was alternated between -90 mV and -50 mV. Also the steps were applied between 5 and 15 minutes after rupturing the membrane patch. I_Ca,L _was measured as inward current during depolarizing 300 ms steps applied from the holding potential of -50 mV in 10 mV increments up to +60 mV. I_Ca,T _was separated from I_Ca,L _by subtraction of currents elicited by 300 ms depolarizing steps from holding potentials of -50 and of -90 mV in increments of 10-mV up to +60 mV. The calcium currents were measured as the difference between the inward peak and the current remaining at the end of the voltage step.

The background current I_K1 _was measured during steps from holding potential of -40 mV in 10 mV increments to test potentials ranging from -20 to -120 mV at a frequency of 0.1 Hz. The holding potential of -40 mV was used to inactivate the sodium channel. For measurement of Na^+^-Ca^2+ ^exchange current (NCX), the external solution (in mM) consisted of NaCl 140, CaCl_2 _2, MgCl_2 _1, HEPES 5, and glucose 10 with a pH of 7.4, and contained strophanthidin (4 μM) nitredipine (10 μM) and niflumic acid (15μM). Test steps were applied from a holding potential of -40 mV to test potentials of -100, -80, -60, -40, -20, 0, +20, +40, +60, +80 and +100 mV.

### Statistics

All quantitative data are expressed as mean ± S.E.M. The differences between healthy and myopathic cardiomyocytes were analyzed by one-way ANOVA. The χ^2 ^test with Yates' correction of Fisher's exact test was used for the categorical data. A value of p < 0.05 was considered to be statistically significant.

## Results

### Action potential characteristics

**Figure 1:**
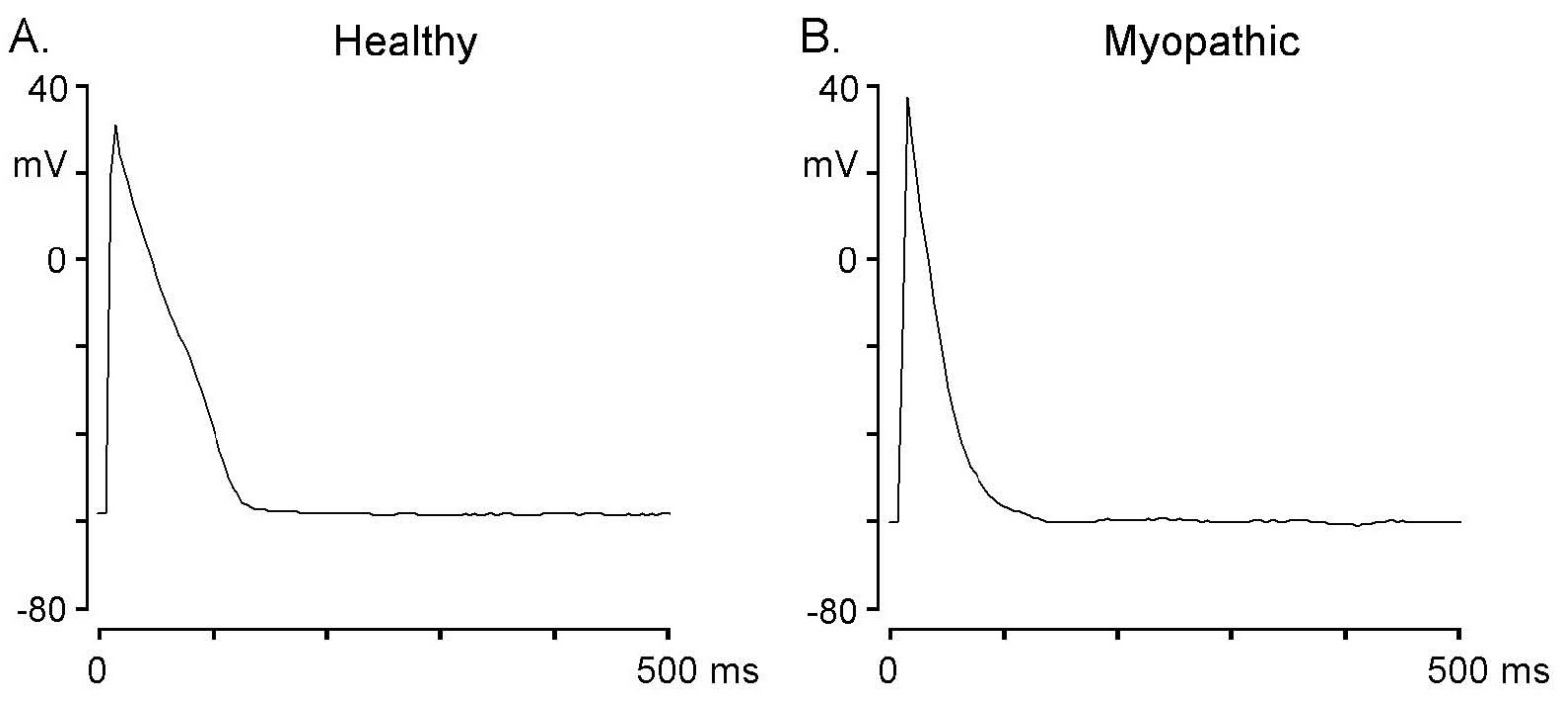
**Different configuration and duration of driven action potentials in LA-PV cardiomyocytes obtained from healthy (panel A) and myopathic (panel B) hamsters**.

In driven cardiomyocytes, APD_50 _and APD_90 _were shorter in myopathic (n = 29) than in healthy hamsters (n = 42). Figure 1A and 1B show typical examples. The difference in APD between the two types of cells was statistically significant (Table 1). Myopathic myocytes had a larger capacitance (Table 1) and thus a larger cell size. Other AP parameters (APA and MDP) were not significantly different between the two groups.

**Table 1:** Electrophysiological properties of healthy and myopathic LA-PV cardiomyocytes isolated from 18 healthy and 17 myopathic Syrian hamsters

	Myopathic	Healthy
Cm(pF)	47 ± 3*(67)	33 ± 2 (83)
APA (mV)	81 ± 3 (29)	87 ± 2 (42)
Phase-0 (mV/ms)	14.1 ± 2.1*(25)	24.2 ± 1.9(34)
MDP (-mV)	60 ± 1 (29)	57 ± 1 (42)
APD_20 _(msec)	21 ± 3 (29)	26 ± 3 (42)
APD_50 _(msec)	40 ± 5* (29)	64 ± 9 (42)
APD_90 _(msec)	77 ± 8* (29)	109 ± 11 (42)

Cm, membrane capacitance of myocyte. For action potential parameters: Steady-state (driven at 1 Hz) values are given in mean ± S.E.M. APA, action potential amplitude. Phase-0  (mV/ms), maximum dV/dt of phase-0 depolarization. MDP, maximum diastolic potential; APD_20_, APD_50_, and APD_90_: action potential duration at 20%, 50% and 90%, respectively, of repolarization levels. Number in parenthesis indicates number of myocytes. *P < 0.05 by group comparisons between healthy and myopathic hamsters.

**Figure 2:**
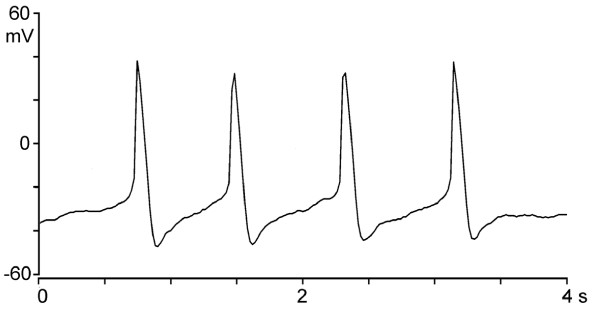
**Spontaneously discharging action potentials in the absence of electrical stimulation recorded in a LA-PV cardiomyocyte**.

In the absence of electric stimulation, the LA-PV cardiomyocytes could be spontaneously active. As illustrated in Figure 2, spontaneous APs had a slow rate of rise of the upstroke and a conspicuous diastolic depolarization, similar to those of pacemaker cells in the sinoatrial node (SA node). Spontaneously active cardiomyocytes were present in both myopathic and healthy LA-PV areas, but they were more numerous in myopathic hamsters (9/29) than in healthy hamsters (4/42) (χ^2 ^= 3.966, p < 0.05).

### Early (EAD) and late (DAD) afterdepolarizations

**Figure 3:**
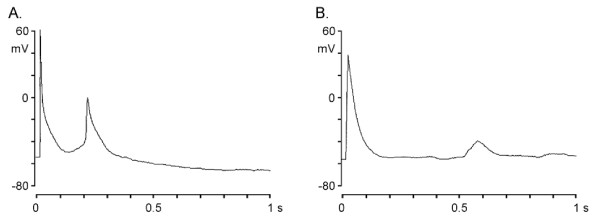
**Triggered rhythms in hamster LA-PV cardiomyocyte. Panel A and B show typical examples of early and delayed afterdepolarization (EAD and DAD, respectively) recorded in a myopathic myocyte stimulated at a rate of 1 Hz in normal Tyrode's solution**.

Triggered rhythms (EAD and DAD) in myopathic LA-PV cardiomyocytes driven at 1 Hz are shown in Figure 3. EAD were present in 5 out of 29 (17%) myopathic and in 4 out of 42 (10%) healthy LA-PV cardiomyocytes. The incidence of DAD was 2/29 (7%) and 8/42 (19%) in myopathic and healthy myocytes, respectively. There were no significant differences in the incidence of EAD and DAD between the two groups. However, it is not readily apparent why EADs and DADs should be present in both normal and myopathic cells.

### L- and T-type Ca current (I_Ca,L_, I_Ca,T_)

**Figure 4:**
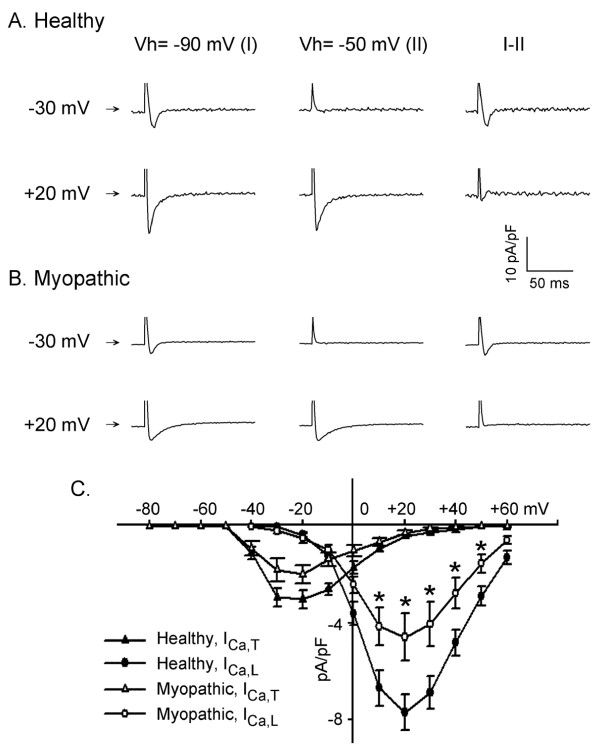
**Differences in two types of calcium currents recorded from healthy and myopathic LA-PV cardiomyocytes**. A and B: selected membrane currents elicited by depolarizing clamps to various voltages from the holding potentials (Vh) of -90 mV (I) and -50 mV (II, I_Ca,L_). Horizontal arrows near left margin indicate zero current. The difference current (I-II, I_Ca,T_) was obtained from the subtraction of current elicited from -90 mV and -50 mV, respectively. C: Mean current densities of peak I_Ca,L _and I_Ca,T _from 10 myopathic and 12 healthy LA-PV myocytes. *P < 0.05 by group comparisons.

Selected membrane current traces elicited by depolarizing steps from V_h _-90 mV (I) and -50 mV (II, I_Ca,L_) in healthy (panel A) and myopathic (panel B) LA-PV myocytes are illustrated in Figure 4. The difference current (I-II, I_Ca,T_) arise from the subtraction of current elicited from V_h _-90 mV and -50 mV, respectively.

The voltage-dependence of peak I_Ca,L _and I_Ca,T _density from 12 healthy and 10 myopathic LA-PV myocytes is shown in panel C. The current-voltage relationship of I_Ca,T _had a threshold voltage of about -40 mV and a peak at -20 mV. The current-voltage relationship of I_Ca,L _had a threshold voltage about -20 mV and a peak at +20 mV. I_Ca,T _was activated at more negative voltage than I_Ca,L _and its density was three-fold smaller than that of I_Ca,L_. The result shows that myopathic myocytes had smaller I_Ca,L _than healthy myocytes (-4.6 ± 0.98 versus -7.8 ± 0.75 pA/pF, p < 0.05). The I_CaT _was also appeared smaller in myopathic myocytes but the difference between myopathic vs. healthy was not statistically significant.

### Transient outward K current (I_TO_)

**Figure 5:**
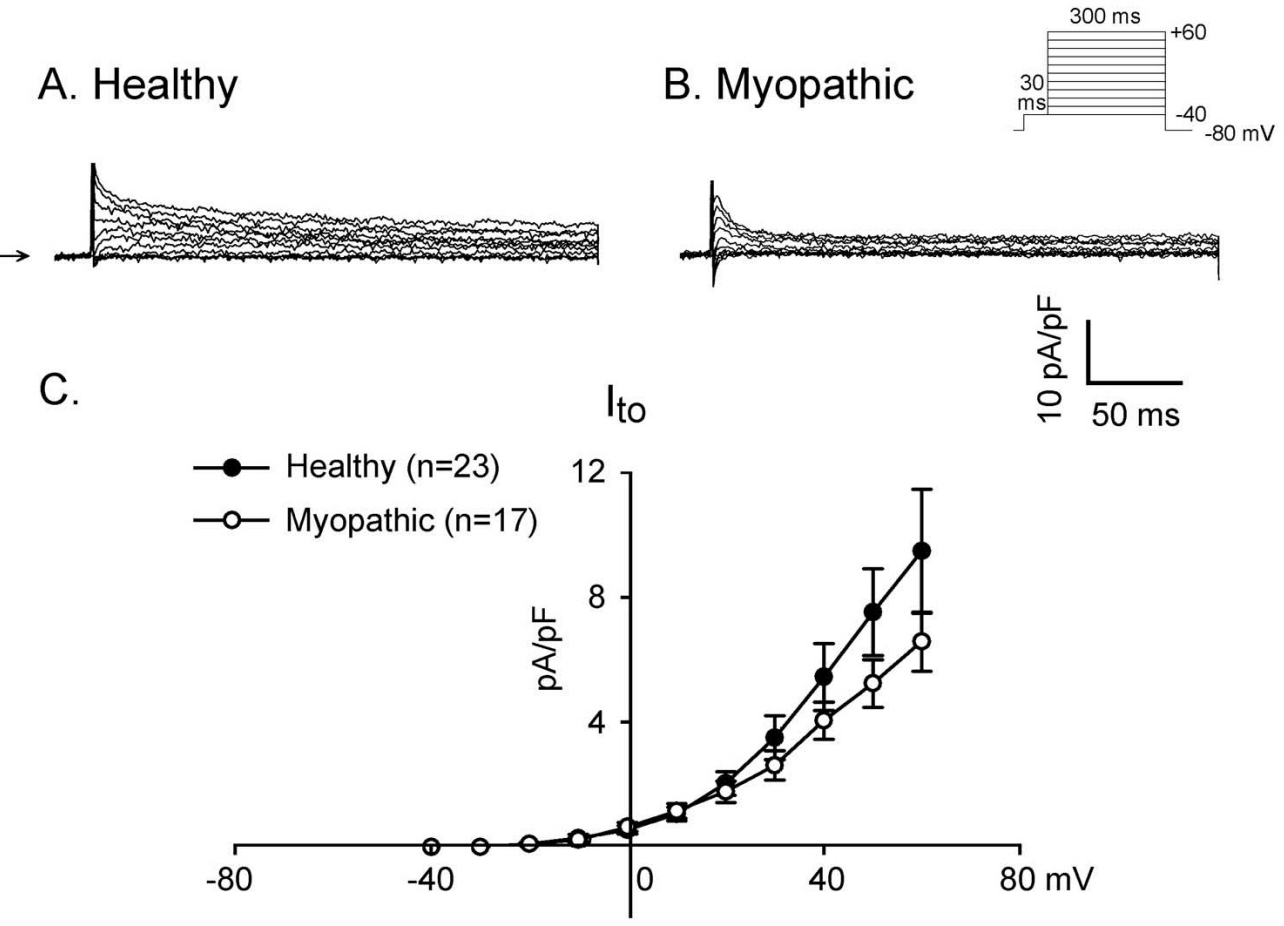
**Transient outward K^+ ^current (I_TO_) activated on depolarization in healthy (panel A) and myopathic LA-PV myocytes (panel B)**. Clamp protocol is shown on top of panel B. The mean current-voltage relations of I_TO _in 23 healthy (closed circles) and 17 myopathic myocytes (open circles) are shown in panel C.

I_TO _was studied with a double-pulse protocol (Figure 5). A 30-ms prepulse from -80 to -40 mV was used to inactivate the sodium channel, followed by a 300-ms test pulse to +60 mV in 10-mV increments. Selected membrane currents in healthy (panel A) and myopathic (panel B) LA-PV myocytes are illustrated in Figure 5. There were no statistically significant differences in voltage-dependent properties of I_TO _in myopathic versus healthy hamsters.

### Delayed rectifier K current (I_K_)

**Figure 6:**
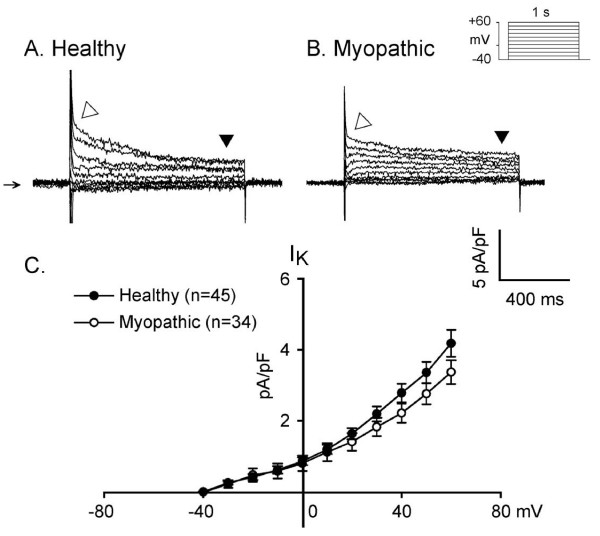
**Delayed outward K^+ ^current (I_K_) activated on depolarization in LA-PV healthy (panel A) and myopathic myocytes (panel B)**. Clamp protocol is shown at the top of panel B. I_K _was measured near the end of the 1 s depolarizing pulses. Downward solid triangle indicated I_K _recorded at test potential of +60 mV. In contrast, open triangle indicated I_TO _in the same myocytes. Panel C shows the mean current-voltage relations of I_K _in 45 healthy (closed circles) and 34 myopathic myocytes (open circles).

Long (1 s) depolarizing steps from V_h _-40 to +60 mV in 10 mV increments (see protocol on Figure 6) induced a slowly activating and non-inactivating outward current, consistent with the behavior of delayed rectified K^+ ^current in hamster LA-PV myocytes. In addition, there was I_TO _with rapid activation kinetics. The voltage-dependence of I_K _was comparable in myopathic and healthy hamsters (Figure 6C).

### Inward rectifier K current (I_K1_)

**Figure 7:**
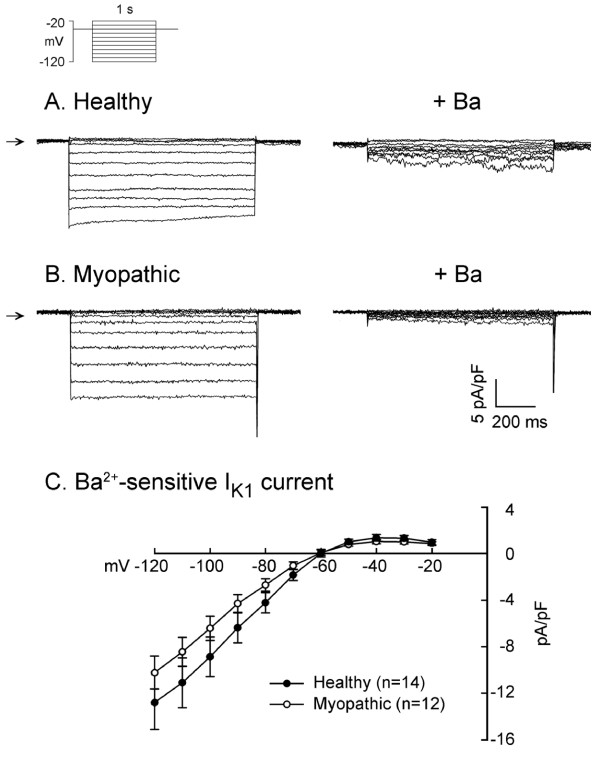
**Ba^2+^-sensitive inward rectifier K^+ ^current, I_K1_**. Panel A illustrates a series of I_K1 _elicited on hyperpolarization before (control) and after 1 mM Ba^2+ ^superfusion in a healthy myocyte. Similar traces obtained from a myopathic myocyte are shown in panel B. Horizontal arrows near left margin indicate zero current. The difference current densities (Ba^2+^-sensitive K^+ ^current, I_K1_) (mean ± S.E.M.) in 14 healthy myocytes and 12 myopathic myocytes are summarized in the current-voltage relations shown in panel C.

I_K1 _was measured in the absence and presence of 1 mmol/L Ba^2+ ^as illustrated in Figure 7. The V_h _was -40 mV and then I_K1 _was measured at test potentials over the range from -20 down to -120 mV (see clamp protocol on top of Figure 7). The difference in the magnitude of I_K1 _in control solution and solution containing Ba^2+ ^gave the Ba^2+^-sensitive inward rectifier K currents. As shown in Figure 7 panel C, the mean Ba^2+^-sensitive I_K1 _was not significantly different between the two groups.

### Na^+^-Ca^2+ ^exchange current (I_NCX_)

**Figure 8:**
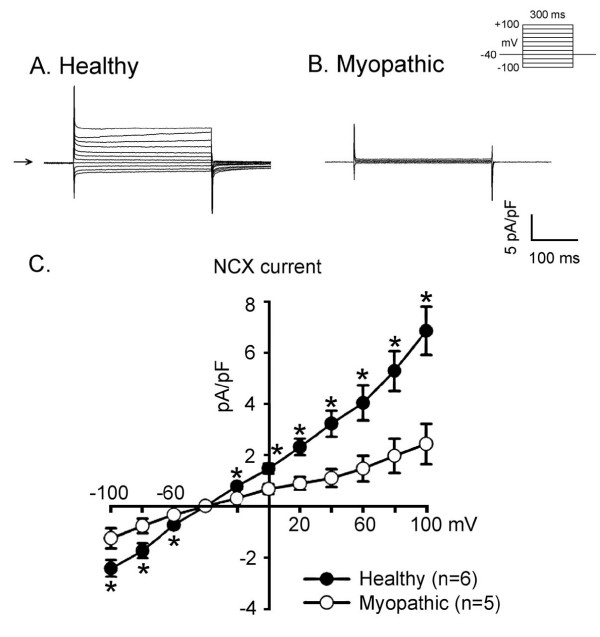
**Na^+^-Ca^2+ ^exchange current (NCX) in healthy (panel A) and myopathic LA-PV myocytes (panel B)**. The clamp protocol is shown between panels A and B. The holding potential was -40 mV. Horizontal arrow at left margin indicates zero current. For inward NCX currents the myocyte was hyperpolarized V_h _-40 to -100 mV in 20 mV increments. For outward NCX currents, the myocyte was depolarized from -40 to +100 mV in 20 mV increments. Mean current-voltage relationships of NCX in 6 healthy and 5 myopathic myocytes (from 3 hamsters in each group) are summarized in panel C.

The protocol tested is shown in Figure 8 (top). In Figure 8 V_h _was -40 mV to inactivate fast Na channel. For inward NCX currents the cardiomyocytes were hyperpolarized to -100~ -60 mV in 20 mV steps from a V_h _of -40 mV. For outward NCX currents, the myocytes were depolarized from V_h _-40 to +100 mV in 20 mV increments. Both the inward and outward I_NCX _were smaller in the myopathic (panel B) than in the healthy cells (panel A). As shown in the current-voltage relationship of I_NCX _(Figure 8, panel C), the difference between the mean outward and inward NCX currents in myopathic cells (n = 5) was significantly smaller than in healthy myocytes (n = 6)

## Discussion

The present results show that the LA-PV cardiomyocytes obtained from the myopathic hamsters at mid- and late-stage of cardiac hypertrophy and dilatation (36-57 week-old) have higher percentage of spontaneously active cardiomyocytes than control myocytes from healthy hamsters. When driven at constant rate, the myopathic myocytes have shorter APD. Also, I_Ca,L _and I_NCX _are smaller in myopathic than in healthy control and this might account for the shorter APD. The currents I_TO_, I_K_, I_K1 _and I_Ca,T _were not significantly different in the two groups. Our results suggest that LA-PV cardiomyocytes of myopathic hamsters have more frequently automatic discharge, but smaller I_Ca,L _and I_NCX _with a shorter APD. The altered electrophysiological properties might favor the occurrence of reentrant rhythms [6,16].

### Automatic rhythms

It has been demonstrated in human myocardium that there are myocardial cells in the junction between atrium and PV [17,18]. In isolated guinea-pig PV tissues, infusion of noradrenaline (10^-7 ^g/ml) can induce spontaneous tachyarrhythmias [19]. Our recent study on PV tissues of dog [1,11] and rabbit [12,13] also demonstrated that PV myocardial sleeves have some cells that can be spontaneously active. In the present experiments, myocytes of myopathic hamsters had automatic activity that was significantly more frequent (31%) than those from healthy hamster (10%,). Thus, the myopathic PVs are more prone to develop automatic arrhythmias.

The increase in automaticity of the myopathic PV cardiomyocytes could be related to one or more of the following ionic mechanisms: an increased I_Ca,T _[20], an enhanced decay in I_K _during diastole, a positive shift of the diastolic potential or a negative shift of the threshold potential or a shorter APD [6,16]. The present electrophysiological results suggest that the significant shorter APD (and presumably a shorter refractory period) with an increased slope of diastolic depolarization may be the most likely factors in the abnormal rhythms.

### Triggered rhythms

Myopathic PV cardiomyocytes tend to develop EAD and DAD which could initiate repetitive discharge. Yet, the incidences of triggered activities in myopathic myocytes were not significantly higher than those of healthy PV cardiomyocytes. Arrhythmias in myopathic myocytes could also be related to the significant shorter APD_50 _and APD_90 _(Table 1), which could facilitate re-entry rhythms through a shorter refractory period. The shorter APs could be related to the smaller calcium currents.

The significantly smaller I_Ca,L _could result in a smaller intracellular Ca^2+ ^concentration ([Ca^2+^]_i_) in myopathic myocytes and therefore be less likely to generate [Ca^2+^]_i _overload and transient inward curren I_TI _on repolarization from depolarizing steps [6,21]. On the other hand, release of Ca^2+ ^from the SR could inactivate the L-type Ca^2+ ^channels [22] thus resulting in a smaller I_Ca,L_. Further experiments using fluorescent method to measure [Ca^2+^]_i _and using whole-cell patch-clamp technique to determine I_TI _in myopathic vs. healthy cardiomyoyctes need be carried out to clarify this point.

### Reentrant rhythms

Hano et al. [23] reported that spontaneous and sporadic ventricular premature contractions (VPC) occurred in 8.3% of Bio14.6 strain cardiomyopathic hamsters, whereas no ventricular arrhythmia was recorded in normal hamsters. Either non-sustained ventricular tachycardia (NSVT) or ventricular fibrillation could be induced in all cardiomyopathic hamsters. In contrast, neither NSVT nor ventricular fibrillation was induced in normal hamsters [23].

Cold-immobilization of Bio14.6 myopathic hamsters for 2 hr had a lethal effect [24]. There was an obvious heart rate slowing and occasional AF or atrio-ventricular block. In contrast, no ill effects were observed in healthy hamsters subjected to similar stress. Propranolol (but not phentolamine or atropine) prevented the lethal effects of the stress. Higher incidence of automatic activity in the presence of shorter APD and/or refractory period could lead to generation of ventricular arrhythmias [23] but could not explain the atrial arrhythmias [24]. Sakamoto [25] summarized the electrical and ionic abnormalities in the heart of cardiomyopathic hamsters and noted the contradictory results from different or even from the same laboratory. Species difference and ages of animals should be considered in the interpretation of the experimental results. Also, reduced coupling of the myocytes in the PV due to histological changes in extracellular collagen matrix in chronic atrial pacing- induced AF may provide an additional mechanism facilitating repetitive rapid activities [26].

### Similarity and difference in arrhythmogenic mechanisms in myopathic hamster versus Xinα-deficient mouse model

We have recently reported a study on the mechanisms of arrhythmias in the LA-PV myocardium of mXin-deficient mice induced by brief high frequency electrical drive (30 Hz for 3 sec) [27] in the absence and presence of isoproterenol, strophanthidin and atropine. It was found that automatic rhythms, triggered rhythms and induction of reentrant AF were affected differently by *mXinα *gene deletion as detected by a MED64 multi-electrode array system [27]. The induction of AF was consistently hindered in mXinα-deficient LA-PV preparations even under conditions that enhance its induction in mXinα+/+ preparations. The mechanisms that prevent the induction of AF in the mXinα-/- preparation appear to involve a decrease in conduction velocity and longer APs: these changes apparently prevent the establishment of reentry paths [27]. However, automatic and triggered rhythms were not suppressed in mXinα-/- preparations.

In contrast to murine cardiomyocytes, the APD of LA-PV cardiomyocytes of myopathic hamster was significantly shorter than that of healthy control (see Figure 2 and Table 1). A shorter AP may favor the generation of reentrant rhythms. Since the incidence of triggered activities in myopathic myocytes was not significantly higher than that in healthy control, other factors such as age-related changes in Na^+^-Ca^2+ ^exchanger have been considered [28,29]. However, the present results show that both inward and outward Na^+^-Ca^2+ ^exchange current was reduced in the LA-PV cardiomyocytes of myopathic hamsters (Figure 8). Therefore, I_NCX _is unlikely to contribute to enhanced automatic rhythms in Bio14.6 hamsters. Further experiments are required to clarify the underlying mechanisms responsible for the arrhythogenesis in myopathic Bio14.6 hamsters.

## List of abbreviations

APD: action potential duration; Bio14.6 and F1B: Syrian hamsters strains Bio14.6 and F1B; LA: left atrium; PVs: pulmonary veins; AF: atrial fibrillation

## Competing interests

The authors declare that they have no competing interests.

## Authors' contributions

YXL: performed most of the experimental works *except *study on NCX current and data analysis. KHW: performed the experimental works on NCX currents and data analysis. YCC: designed the experimental protocols for measurements of ionic currents and general instructions for patch-clamp study. CHH: discussion on experimental data (especially those for NCX currents), grant support for the experimental study. JW: general discussion on the experimental works and grant support for the study. CIL: principal investigator of the study, research grant applications to NSC, design of the electrophysiological study, data analysis and interpretation and writing up of the manuscript.
